# Food consumption and undernutrition variations among mothers during the post-harvest and lean seasons in Amoron'i Mania Region, Madagascar

**DOI:** 10.1186/s12889-019-7333-9

**Published:** 2019-07-26

**Authors:** Lantonirina Ravaoarisoa, Julio Rakotonirina, Lalhyss Randriamanantsaina, Jean de Dieu Marie Rakotomanga, Michèle Wilmet Dramaix, Philippe Donnen

**Affiliations:** Institut National de Santé Publique et Communautaire Antananarivo Madagascar, Faculté de Médecine d’Antananarivo Madagascar, Ecole de Santé Publique de l’Université Libre de Bruxelles, Brussels, Belgium

**Keywords:** Seasonal effect, Undernutrition, Food consumption, Mother, Nutritional status

## Abstract

**Background:**

Seasonal variation affects nutrition particularly in contexts where people’s food consumption depends on their production of food. Assessing the effect of the season on nutrition status can help us to identify strategies to address undernutrition. This study aims to measure the variations in food consumption and the incidence of undernutrition according to season, and to identify the factors associated with the incidence of undernutrition.

**Methods:**

A cohort study was conducted among 608 mothers aged between 18 and 45 years living in the Amoron’i Mania Region of Madagascar. Inclusion in the study occurred during the post-harvest season, and mothers were followed until the end of the next lean period (7 months). A dichotomous variable of the frequency of consumption of various foods was used to establish variation in food consumption. Body Mass Index < 18.5 kg/m^2^ and Middle Upper Arm Circumference < 220 mm were used to measure incidence of undernutrition. A generalized linear model was used to identify factors associated with the incidence of undernutrition and to derive relative risks.

**Results:**

During the lean season, the frequency of consumption of leafy green vegetables, peanuts, fish, and eggs decreased significantly. In contrast, the frequency of fruit, legumes, and non-leafy green vegetables consumption increased significantly. The prevalence of undernutrition (based on the BMI and/or MUAC) among mothers increased from 19.6% in the post-harvest period to 27.1% in the lean period (*p* < 0.001). The incidence of undernutrition (based on the BMI and/or MUAC) during the follow-up was 12.2%. The factors related to undernutrition were low and medium score of movable property possession (Adjusted RR = 3.26 [1.33–7.94] and Adjusted RR = 2.48 [1.01–6.10]), no toilet (Adjusted RR = 1.76 [1.07–2.91]), and pregnancy (Adjusted RR = 2.92 [1.42–6.04]) (based on the MUAC only for pregnancy).

**Conclusion:**

This study highlights the variation in the frequency and type of food consumption and subsequent deterioration in mothers’ nutritional status during the lean season. Economic, hygiene, and reproductive factors were associated with undernutrition. Analyzing the existing interventions to fight maternal undernutrition is necessary to determine whether or not seasonality is considered and addressed.

## Background

The relationship between the season and the nutritional status of the population has long been recognized as relevant. The season has known effects on food availability, food consumption, and population nutrition status in high, middle, and low-income countries [1–6]. The effect of seasonal variation on nutrition is particularly important in contexts where people’s consumption depends on their agricultural production [1, 7, 8]. In low- and middle-income countries, the adult workload and associated energy expenditure change according to the season and have an effect on nutritional status. Subsistence farming is undertaken almost entirely by the farmers themselves, and their workloads are heavier during the growing season. In Africa, women account for 60% of food production, from working the land to harvesting and selling products [9]. Moreover, women are more vulnerable to undernutrition, and they are thus more vulnerable to the negative effects of these seasonal variations.

In Madagascar, 73% of households are farming households that mainly practice subsistence farming [10]. The country has a high prevalence of undernutrition, which has not improved for several years despite programs to address it. While the prevalence of undernutrition (Body Mass Index, BMI < 18.5 kg/m^2^) among women has decreased in Africa and Asia since 1980, it has increased in Madagascar from 19 to 27% between 2003 and 2009 [11–14]. Interventions to address undernutrition seemingly do not take into account this seasonal effect, in part because there is no extant data on the association between season and undernutrition, and the factors moderating this association. Such data can help program planners to better address undernutrition. Thus, the objective of this study was to measure the variations in food consumption, to measure the incidence of undernutrition of mothers according to the season, and to identify the factors associated with the incidence of undernutrition.

## Methods

### Study site

The study was conducted in the Amoron’i Mania Region, one of Madagascar’s 22 regions. The region comprises 4 health districts and 53 communes (52 of which are located in rural areas) and has a total population of about 580,000 inhabitants. According to the most recent Demographic Health Survey (DHS), which was undertaken in 2008–2009, this region has the highest prevalence of undernutrition (BMI < 18.5) among women of reproductive age in Madagascar. The DHS found the prevalence of undernutrition to be 41.6% among women of reproductive age [14].

Agriculture, mainly subsistence, is the main activity of the population (agricultural household share: 96%). The median amount of land held in the study area is 0.4 ha per farming household. Rice remains the most widely cultivated crop, followed by cassava and potatoes. Rice is the staple food of the majority of the population and the harvest season corresponds to the rice harvest. In 2013, 86% of the population was living below the national poverty line (< 1.1$ Purchasing Power Parity, PPP) [10, 14].

### Study population

A cohort study was carried out among mothers. Inclusion in the study occurred in July 2015 in the post-harvest season and mothers were followed until the end of the next lean season in February 2016. The study population included mothers between 18 and 45 years of age, non-pregnant (checked by a pregnancy test strip), and who had given birth more than 6 months earlier. Women who became pregnant during the follow up were not excluded. Mothers under 18 years old were not included because of the difficulty of obtaining the guardian’s consent. Mothers over 45 years old were not included so that the study would focus on mothers in their most fertile reproductive period, as the fertility rate is very low for women between 45 and 49 years old [14]. To ensure the validity of weight measurements, we also excluded women who had given birth within the last 6 months. Mothers’ weight stabilizes around the sixth month after deliveries [15, 16].

**Note:** a baseline analysis was performed with the data obtained at the beginning of the study (post-harvest season) and the results has been published previously (“Socioeconomic determinants of malnutrition among mothers in the Amoron’i Mania region of Madagascar: a cross-sectional study” BMC Nutrition (2018) 10.1186/s40795-018-0212-4). Thereby, this methodology, especially the study population, the sampling and the data collection, has been published and provide the attribution to the source mentioned above.

### Sampling

The sample size was calculated for the baseline cross-sectional analysis that was carried out at the beginning of this study. Sample size was calculated on the basis of the national prevalence of undernutrition (27%), 5% margin of error, 95% confidence level, and a design effect of 2 [17]. The required sample size was estimated to 606. The sample size was computed to reach an adequate precision for malnutrition prevalence. Post-hoc analysis indicates that the study has a high power (99.6%) for the comparisons between undernutrition proportions in the two seasons.

A two-stage cluster sampling was used. The first stage aimed at selecting 30 “fokontany” (smallest administrative structure) out of the 760 in the region. It was done by systematic random sampling. The second stage was used to select by simple random, in each “fokontany”, eligible mothers from an exhaustive list established by community workers. Twenty-one subjects per cluster therefore had to be included. During data collection, 670 women were actually interviewed.

### Data collection

Data collection was conducted in the post-harvest season (July–August 2015) and in the lean season (February 2016). The harvest season corresponds to the rice harvest, which is the staple food of the population of the study area. The annual number of rice harvest periods varies by locality depending on the climate and soil fertility (1 to 2 times per year). In most of the Amoron’i Mania area, rice harvesting occurs once a year (March–May). During the lean period (November–February), the population has difficulty in obtaining food because annual rice production does yield an adequate supply for the entire year.

Information about the socio-economic profile was collected at the beginning of the study by interview. The information about mothers’ nutritional status and their food consumption was collected at the beginning (post-harvest season) and at the end (lean season) of the follow-up. Nutritional status was assessed by anthropometric measurements including weight, height and Mid Upper Arm Circumference (MUAC). Women were weighed within 100 g accuracy using a SECA electronic scale. Height was measured with 1 cm accuracy with a SECA wall mounted height rod. MUAC was measured on the left arm, midway between the acromion and the olecranon, with 1 mm accuracy with an adult-specific measuring tape.

Food consumption was assessed by a food frequency questionnaire created by the team, which included questions about the 3 months preceding the survey. This rather extended period was chosen to provide the study team with information about diets during the harvest and lean seasons and about rarely consumed food. The list of foods for which frequency of consumption was requested was based on two sources: the standard list of foods used to calculate the dietary diversity score for women in low-income countries and the variety of foods typically consumed by the population identified in an exploratory study [18, 19].

Interviewers were recruited locally. All interviewers had at least a bachelor’s degree and were fluent in the local dialect. They were trained to administer the tools created for this study. The investigator, a technician from the Nutrition Department of the Ministry of Public Health, and the Regional Manager for Nutrition supervised the data collection. The study was approved by the Malagasy Ministry of Health Ethics Committee.

### Variables of the study

#### Dependent variables

- Food consumption frequency: this variable was categorized into 2 groups: no or less than once a week consumption and consumption once or more than once a week. Difference of food frequency consumption between post-harvest period and lean period was used to asses effect of season in dietary.
- Undernutrition: BMI and MUAC were used to define undernutrition. The BMI was calculated by dividing weight in kilograms by height in square meters. WHO defined standards were used to identify undernourished women, i.e. BMI below 18.5 kg/m^2^ and we chose a cut-off of 220 mm for MUAC [15, 20]. We did not analyze BMI for mothers who became pregnant at the end of the study because there is no reference value to identify undernourishment for them [20]. Prevalence and incidence of undernutrition were used to analyze effect of season.

#### Independent variables

Information about age, education level (highest level of education completed), occupation, and marital status were collected. Information about parity, number of children aged under 5, breastfeeding, birth interval, and use of contraceptive method were also collected. The birth interval was calculated for the last two deliveries (live birth) within the last 5 years. Afterwards, that interval was grouped using a 24-month threshold. Mothers who had not had two childbirths within the last 5 years were classified in the 24 months or more group.

We also collected data on household-related variables, namely the gender of the head of household, the quality of the water used for food preparation, the toilet type, the fuel used, and the house type. The water source was considered to be improved if it came from standpipes, public faucets, covered or protected wells, wells, boreholes with pumps, and faucets inside or outside the dwelling.

The indicator of economic level was created considering the possession of movable property (furniture, radio, TV, bicycle, etc.). The corresponding scores for these properties were established by principal component analysis (PCA). The score was categorized into three groups (high, medium and low) based on values as close as possible to the tertiles. The period (number of months per year) in which a household consumed its rice production was also collected. Rice production is considered to be an essential element of food security in the study area, such that it can be a partial proxy for economic status.

### Data analysis

Stata / IC 13.1 (StataCorp LP, College Station, USA) software was used to analyze the data.

Proportions of food consumption frequency more than once a week in the two periods (post-harvest and lean season) were calculated and compared for each food/foods group. A McNemar test was used to compare these proportions.

Prevalence of undernutrition in the two periods was calculated according to BMI < 18.5 kg/m^2^, to MUAC< 220 mm, and to BMI and/or MUAC. McNemar test was used to compare the prevalence of undernutrition in the two periods.

Undernutrition incidence during the follow-up was calculated and analyzed. The association between maternal undernutrition incidence during follow-up and each of the other variables was assessed using chi-square tests and by computing crude Relative Risks (RR) with a 95% Confidence Interval (95%CI). To obtain adjusted RR, we used a multivariable Generalized Linear Model (GLM) with log link and binomial distribution. The variables included in the model were selected on the basis of our hypotheses. We assumed that mothers’ reproductive history, hygiene, and socio-economic variables would predict incidence of malnutrition. So, we choose to introduce the following in the model: movable property score, education level, household size, birth interval, and possession of toilets. A link test was performed for model specification. The link between pregnancy and the incidence of undernutrition was analyzed separately. Categorical variables with more than two categories were transformed into indicators. The significance level (*p*-value) was set at 0.05.

## Results

A total of 670 mothers were included in the study of which 62, or 9.3%, were lost to follow-up. Most of those lost to follow-up no longer lived in the area (*n* = 52), 4 mothers gave birth (premature delivery) and 6 mothers refused to continue. Among the 608 mothers present at the end of the study, 55 were pregnant.

### Sample description

Table 1 shows the characteristics of mothers and their households. Mothers’ age ranged from 18 to 45 years, and 14.6% of them were illiterate. Agriculture was the main economic activity the mothers engaged in. At the beginning of the follow-up, 41.8% of mothers were breastfeeding and 30.4% were at the end. The mean household size was 6, with a standard deviation of 2. Most of mothers’ households (83%) draw water from unimproved sources.

**Table 1:** Description of mothers by socio-economic profile and household characteristics at the beginning of the study

Characteristic	n	%
Age mean (year) (SD)	608	33 (7)
Educational status		
Illiterate & Primary	455	73.2
Secondary 1^rst^ cycle	134	22.0
Secondary 2nd cycle	29	4.8
Occupation		
Farmer	550	90.5
Others	58	9.5
Live in couples		
Yes	467	76.8
No	141	23.2
Parity		
1–3	264	43.4
4–5	182	29.9
6 and +	162	26.7
Nb children < 5 years		
0	167	27.1
1	250	41.1
2	161	26.5
3–4	32	5.3
Interbirth interval (month)		
< 24	86	14.1
≥ 24	522	85.9
Breastfeeding		
Yes	252	41.8
No	354	58.2
Use contraceptive method		
Yes	275	45.2
No	333	54.8
Household size		
< 6	281	46.2
≥ 6	327	53.8
Head of household		
Male	448	80.3
Female	120	19.7
Water source		
Improved	103	16.9
Unimproved	505	83.1
Have toilet		
Yes	444	73.0
No	164	27.0
Fuel for cooking		
Wood	578	95.1
Others	30	4.9
Traditional house^a^		
Yes	333	54.3
No	275	457
Duration of rice availability		
≤ 6 months/12	413	67.9
> 6 months/12	118	19.4
Non producer	77	12.7

^a^ earthen floor and wall and straw roof

The comparison between those mothers who were lost to follow-up mothers (*n* = 62) and those who were not (*n* = 608) showed that the movable property possession score and toilet possession had significant differences. Lost to follow-up mothers had a higher movable property possession score (50% versus 34%) and were more likely to have a toilet (85% versus 74%). Compared to non-pregnant mothers at the end of follow-up (*n* = 553), pregnant mothers (*n* = 55) had a lower average age (30 years versus 34 years), a higher proportion who lived in couples (95% versus 75%), and came disproportionately from households headed by men (95% versus 79%).

### Food frequency consumption

**Fig. 1:**
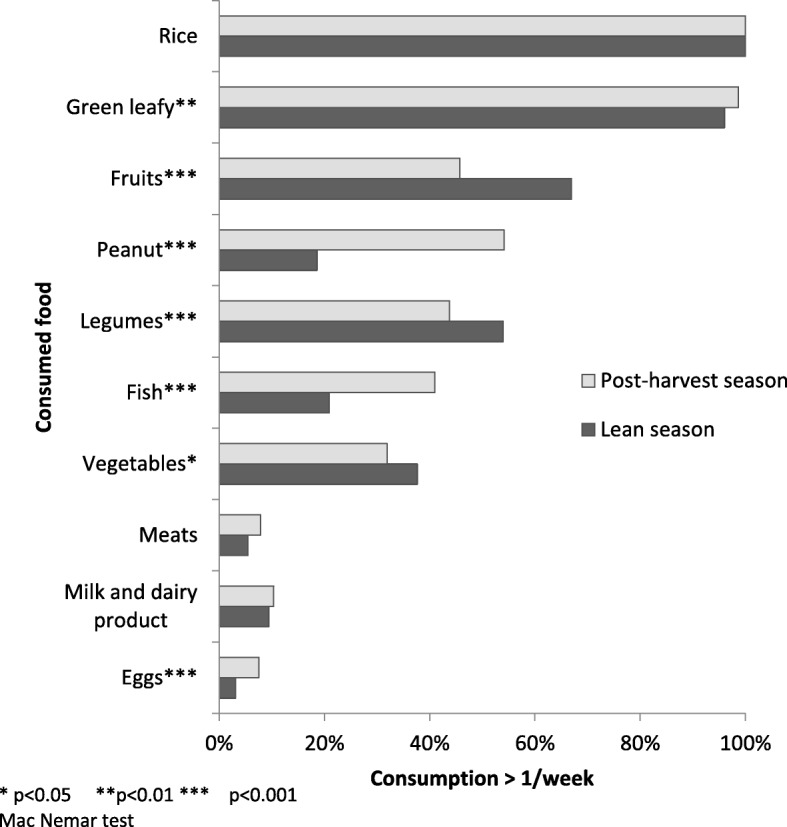
Comparison of proportion of mother who ate food/food group more than once a week in post-harvest and lean season (*n* = 608). Post-harvest season, lean season, rice, green leafy, fruits, peanut, legumes, fish, vegetables, meats, milk and dairy product, eggs

Figure 1 shows the comparison of frequency of particular foods and food group consumption during the two seasons: post-harvest and lean. There was no significant difference in the frequency of consumption of rice, meats, and milk and other dairy products. There were, however, marked differences in the consumption of green leaves, peanuts, fish, and eggs, with the frequency of consumption of these foods decreasing significantly during the lean season. In contrast, the frequency of fruit, legume, and vegetable consumption increased significantly during the lean season.

### Prevalence of undernutrition

**Fig. 2:**
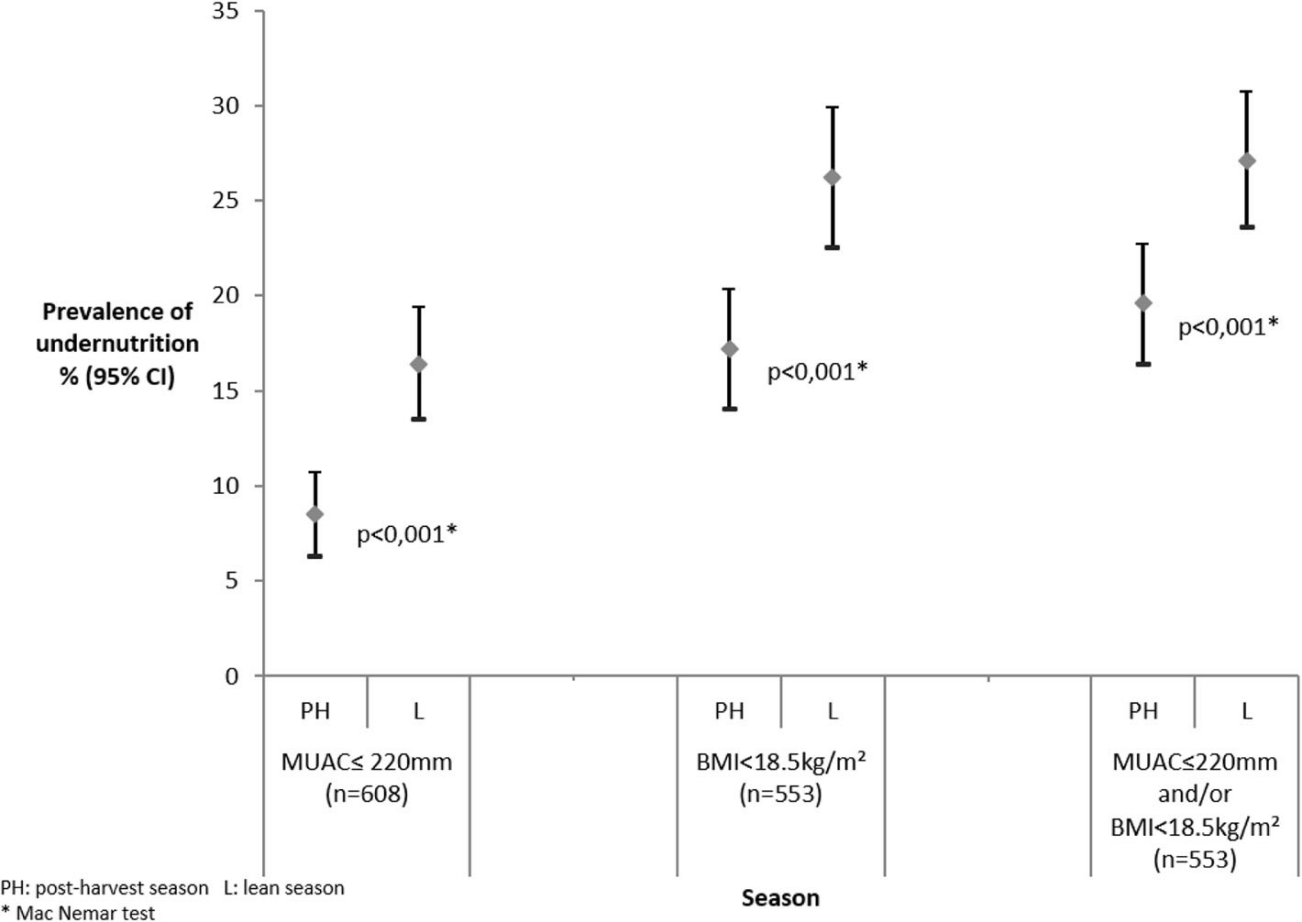
Comparison of the prevalence of under-nutrition in post-harvest and lean season. Post-harvest season, lean season, MUAC≤220 mm, BMI < 18.5 kg/m^2^, MUAC≤220 mm and/or BMI < 18.5 kg/m^2^, % (95%CI)

Based on the MUAC measures (*n* = 608), the prevalence of undernutrition was 8.6% at the beginning of the follow-up (post-harvest period) and 16.5% at the end of the follow-up (lean season) (*p* < 0.001, Mc Nemar test). According to BMI (*n* = 553, pregnant not included), the prevalence of undernutrition was 17.2% in post-harvest period and 26,2% in the lean season (*p* < 0.001, Mc Nemar test). Considering the two indicators (BMI and/or MUAC), 19.6% of mother were undernourished at the beginning of the study (*n* = 553) and 27.1% at the end (*p* < 0.001, Mc Nemar test). Figure 2 describes the prevalence of undernutrition according to the season. For both indicators, the prevalence of undernutrition increased significantly during the lean season.

For the 55 mothers who became pregnant during the follow-up period, 16.4% were undernourished at the beginning of the study and 30.9% at the end (based on the MUAC).

### Incidence of undernutrition

Among the 443 non-pregnant mothers who were well-nourished at the beginning of the follow-up, 54 became undernourished during lean season. The incidence of undernutrition during the follow-up was 12.2% (*p* < 0.001, Mc Nemar test). Among 110 undernourished mothers, 16 (14.5%) became well-nourished at the end of the follow-up.

Compared to non-pregnant women, the incidence of undernutrition (based on MUAC) was significantly higher in pregnant women, adjusted RR = 2.92 [1.42–6.04] (adjusted for variables that differ significantly across the two groups: age, marital status, and gender of the head of household).

Table 2 shows the results of bivariate and multivariate analyses of the incidence of undernutrition for non-pregnant women during the follow-up (Based on the BMI or/and MUAC). In bivariate analysis, the incidence of undernutrition was significantly high for mothers who had a low education level, who had a low or medium movable property score, who did not have a toilet and who lived in a traditional house. The model that explains the variation of incidence of undernutrition for mothers during follow-up shows that the factors that best determine high incidence of undernutrition were low and medium movable property score and the absence of a toilet.

**Table 2:** Associations between incidence of undernutrition with the characteristics of mother and their household

	n	Incidence (BMI and/or MUAC) (%)	Crude RR (95%IC)	Adjusted RR (95%IC)
Educational status^a^				
Low	322	15.2	3.68 (1.50–9.02)	0.43 (0.17–1.08)
High	121	4.1	1	1
Birth interval (month)				
< 24	65	10.8	1	1
≥ 24	378	12.4	1.15 (0.55–2.44)	1.50 (0.72–3.13)
Household size				
< 6	214	10.8	1	1
≥ 6	229	13.5	1.26 (0.76–2.09)	1.19 (0.73–1.93)
Movable property possession score				
Low	147	19.7	4.87 (2.08–11.37)	3. 26 (1.33–7.94)
Medium	148	12.8	3.17 (1.30–7.70)	2.48 (1.01–6.10)
High	148	4.1	1	1
Have toilet				
Yes	327	8.9	1	1
No	116	21.6	2.82 (1.57–5.06)	1.76 (1.07–2.91)

^a^Low = Illiterate & Primary; High = Secondary

## Discussion

Our study shows the variation of the frequency of food consumption and of the nutrition status of a representative sample mothers in Amoron’i Mania region. Mothers who were lost to follow-up had a better socio-economic profile (higher movable property score) that could alleviate the difficulties of the lean period leading us to overestimate the incidence of undernutrition (bias away from the null). This variable was included in the final model of multivariate analysis for adjustment. However, the proportion of lost to follow up was relatively low (9%). The absence of similar studies carried out in Madagascar and the specificity of each local context limit the relevance of comparing our results to other studies.

The frequency of food consumption varied according to the season. Most of the women in this study were farmers and their food intake depended mainly on agricultural production. The high availability of fruits, legumes, and vegetables during the lean season explains the frequency of their consumption in this period. The lean season coincided with the production of peaches, apples, mangoes, and pineapples, and the majority of households harvested these crops in their fields. The high availability of fish and peanuts was observed during the post-harvest season. Fish come mainly from rice fields and streams, and their exploitation is permitted only after the rice harvest. Peanuts are the only product rich in fat that the population of the region grows. During the lean season, peanuts are not yet mature, and few households have them in reserve from the previous season.

The variation in food consumption, mainly fruits and vegetables, according to their period of production, has been reported in studies of women farmers in low- and middle-income countries [21–23]. Rice is the primary food staple for the population in the study area and we did not observe any difference in rice consumption between the two seasons. For this study, the frequency of consumption was categorized according to the week (consumption less or more than once a week). Another study in this area found that the frequency of daily rice consumption and the number of times rice was consumed weekly decreases during the lean season [19]. The population in the study area usually eats rice three times a day. A decrease in the frequency of rice consumption reflects a problem of food insecurity [19]. Variations in the frequency of food consumption during the lean season may be accompanied by a decrease in energy intake. This information is not available such as, but the increased undernutrition frequency during that period confirms the hypothesis.

The prevalence of undernutrition (BMI and/or MUAC), increased significantly during the lean season (from 19.6 to 27.2%) and the incidence of undernutrition between the two seasons was high (12.2%). The season has a substantial impact on the nutritional status of mothers in the study area. Other studies report the deterioration of nutritional status of women as depending the season, but its magnitude varies from one context to another. For example, the prevalence of undernutrition (BMI < 18.5) in women with children under the age of 5 increased by 6% during the lean season, according to a study in Burkina Faso, and by 13% for lactating mothers in Ethiopia [24, 25].

The lean season is known for its low availability and accessibility to food. The deterioration of nutritional status during this period corresponds to a negative energy balance caused by a decrease in energy intake and an increase in energy expenditure [26]. In the region studied, the lean period coincided with the weeding work in the fields, which requires significant energy expenditure, and which is carried out primarily by women. Some mothers are compelled to work as day laborers in order to buy foods that are in shortage during the lean period. High activity in agriculture can cause weight loss in women [27]. In low- and middle-income countries, high energy expenditure is observed among women farmers during the growing period [7, 28]. For the population of the studied region who practice subsistence farming, interventions that increase agricultural production would likely help to reduce undernutrition. Such interventions could allow many mothers to eat well, to work less hard, and to remain in good nutritional status throughout the year.

The study identified three factors that were positively associated with undernutrition during follow-up: low/medium movable property score, not having a toilet and pregnancy. Lack of resources and an unhealthy environment represent two important underlying causes of undernutrition [12]. The movable property score reflects the economic level of the mother’s household. Mothers who have a high movable property score likely have more options for coping with the difficulties encountered during the lean season. Unhealthy environments can contribute to diseases that have negative impacts on nutritional status [29, 30]. The absence of a toilet, as identified in this study, adds to the problem of water hygiene, which has been identified as one of the causes of undernutrition among mothers in the studied region [31]. Diseases related to water and environmental insalubrities are common in the studied region (intestinal parasitosis, bilharziasis, malaria, etc.). The deterioration in nutritional status during the lean period in pregnant women show the impact of the reproduction on the nutritional status of women. Being pregnant implies additional nutritional needs that are more difficult to satisfy during the lean season. In the low-income countries, frequent and close pregnancy is one of the causes of undernutrition [32]. Infants who are born are likely to suffer from the adverse effects of their mother’s undernutrition [33, 34]. It is necessary to integrate management of undernourishment for pregnant women during prenatal care. Taking into account the identified factors helps to alleviate the effect of the lean period on the nutritional status of mothers. The study does not claim to have included all the determinants of undernutrition. Oher factors like dietary practice, energy balance, and health status should be taken into account.

## Conclusions

This study highlights the importance of the seasonal variation in food consumption, and the increase in the prevalence and incidence of undernutrition in the lean season. Mothers of a lower economic status, mothers with environmental hygiene problems, and pregnant mothers were identified as the most vulnerable groups to deterioration in nutritional status. It is necessary to analyze existing interventions to determine whether or not seasonal variation is addressed. Taking into account seasonal variation in the fight against maternal undernutrition in this region may significantly reduce the incidence of undernutrition.

## Data Availability

The datasets used and/or analyzed during the current study are available from the corresponding author on reasonable request.

## Abbreviations

AOR: Adjusted Odds Ratio
BMI: Body Mass Index
DHS: Demographic and Health Survey
MUAC: Mid Upper Arm Circumference
PCA: Principal Component Analysis
